# MicroRNA-126-3p Attenuates Intracerebral Hemorrhage-Induced Blood-Brain Barrier Disruption by Regulating VCAM-1 Expression

**DOI:** 10.3389/fnins.2019.00866

**Published:** 2019-08-16

**Authors:** Xi Fu, Tiesheng Niu, Xiaodong Li

**Affiliations:** Department of Cardiology, Shengjing Hospital of China Medical University, Shenyang, China

**Keywords:** intracerebral hemorrhage, miR-126-3p, VCAM-1, BMECs, BBB

## Abstract

**Background:**

miR-126 is closely related to the occurrence of various complications after intracerebral hemorrhage (ICH), but the molecular mechanism is not fully elucidated. This study aimed to explore the mechanism of miR-126-3p in alleviating brain injury after ICH.

**Methods:**

Serum miR-126-3p levels were compared between patients with IHC and healthy controls. A rat model of ICH was generated by intracerebral injection of Type VII collagenase. The rats were intracerebral injected with miR-126-3p mimics or negative control miRNA. Rat brain microvascular endothelial cells (BMECs) were used as a cell model of blood-brain barrier (BBB), and validated by immunofluorescence staining of Factor VIII. The BBB permeability of BMECs after miR-126-3p antagomir transfection was determined by FITC-dextran 20 through a confluent BMECs layer (measured over 120 min). The binding site of miR-126-3p in the 3′UTR of VCAM-1 was predicated by TargetScan, and verified by dual luciferase reporter assay. The expression levels of miR-126-3p and vascular cell adhesion molecule-1 (VCAM-1) in rat brain tissues and BMECs were measured by real-time PCR or western blotting.

**Results:**

Serum miR-126-3p level was markedly down-regulated in patients with ICH. The rats with ICH had decreased miR-126-3p levels in serum and hemorrhagic area, while those changes were reversed by the treatment with miR-126-3p mimic. VCAM-1 is a direct target of miR-126-3p, and VCAM-1 expression in hemorrhagic area was down-regulated by the administration of miR-126-3p mimic in rats. Inhibition of miR-126-3p by anti-miR126 treatment in BMECs resulted in barrier leakage.

**Conclusion:**

miR-126-3p attenuates intracerebral hemorrhage-induced blood-brain barrier disruption, which is associated with down-regulated expression of VCAM-1 in hemorrhagic area.

## Introduction

Intracerebral hemorrhage is a common life-threatening type of stroke (Min et al., 2016). Although efforts have been made to alleviate ICH and post-ICH complications, the outcome is far from being optimal. Approximately 20% of ICH patients have to suffer from remained varying degrees of neurological dysfunction after treatment (Duan et al., 2016).

MicroRNA (miRNA) is a class of endogenous, 18–23 nucleotides non-coding small RNA. Accumulating studies have clarified the origin of circulating miRNAs in serum (do Amaral et al., 2017; Zhou et al., 2018). In brief, miRNAs are transcribed in the nucleus as pri-miRNAs with 5′-caps and 3′-polyA tails. Drosha removes the cap and polyA tail to generate pre-miRNAs, which are exported from the nucleus to the cytoplasm via Exportin 5. In the cytoplasm, pre-miRNAs are cleaved into mature miRNAs by Dicer. Pre-miRNAs and mature miRNAs can excrete from the cells into the bloodstream via binding to RNA-binding proteins, microvesicles, exosomes or multi-vesicular bodies. miR126-3p levels are decreased in the serum of ICH-injured rats may be due to that injured brain tissues expressed lower levels of miR-126-3p and reduced release into blood.

Many studies also have highlighted the roles of miRNAs in regulating the pathogenesis, diagnosis, and treatment of ICH. For examples, Leung et al. (2014) showed that miR-124-3p and miR-16 were elevated in the serum of ICH patients. Moreover, the levels of plasma miR-29c and miR-122 are proposed to be related to hematoma enlargement (Martinez and Peplow, 2017). Previous studies showed that the serum levels of miR-126, miR-146a, miR-let-7a, and miR-26a were significantly down-regulated in ICH patients when compared with healthy controls (Zhu et al., 2015). Since miR-126 has been shown to play a crucial role in vascular integrity and angiogenesis (Wang et al., 2008), we are particularly interested in miR-126. It has been reported that miR-126 weakens leukocyte adherence and vascular inflammation by targeting and binding to vascular cell adhesion molecule-1 (VCAM-1) (Harris et al., 2008). The study from Bai et al. (2017) suggested that miR-126 plays a neuroprotective role in ischemic retinopathy by regulating VCAM-1 and BCL2-like 11. A previous study by our group showed that miR-126-3p attenuates BBB disruption, cerebral edema, and neuronal injury in ICH rat models (Xi et al., 2017). Low expression of miR-126 has been identified to be associated with larger perihematomal edema (Zhu et al., 2015). In addition, the overexpression of miR-126 was shown to protect against ICH complications by increasing the levels of VEGF-A and decreasing caspase-3 in endothelial cells (Kong et al., 2017). Furthermore, through quantifying endothelial-specific miRNAs in cerebral arterioles from wild type mice and from pathological mice models of chronic kidney disease (CKD), a study also identified miR-126 as one of the most dysregulated miRNAs, which suggests that miR-126 is a potential new biomarker of cerebral troubles of CKD patients (Metzinger-Le Meuth et al., 2014). Collectively, these findings suggest that miR-126 helps maintain the integrity of vascular physiological structures and the normal barrier function of endothelial cells. However, the protective role of miR-126 in ICH-induced BBB disruption has not been fully elucidated so far.

The BBB prevents neurotoxic plasma components, blood cells, and pathogens from entering the brain, BBB dysfunction relates to neurological deficits in ICH, stroke, traumatic brain injury, and so on (Chen et al., 2016; Sweeney et al., 2019). The monolayer cell structure formed by BMECs are a major component of the BBB, and they maintain BBB functions in the brains (Abbott et al., 2006). Therefore, BMECs are chosen in our present study. BMECs bind to the basement membrane through connexins, and form NVU with astrocytes, pericytes, and smooth muscle cells (Neuwelt et al., 2011). Brain endothelial cell lines can be used to establish the *in vitro* BBB models. To a certain extent, brain endothelial cell lines are superior to primary BMECs, because of their rapid growth and stability over several generations. Nevertheless, because of the inherent characteristics of the primary brain cells, BMECs cannot be completely replaced by brain endothelial cell lines. Multiple markers are currently used to assist in the identification of BMECs, including endothelial cell markers and tight junction protein (Stebbins et al., 2016; Qian et al., 2017).

In the current study, we explored the method of separating primary BMECs based on the method established by Ruck. (Ruck et al., 2014), and validated the isolated cells from neonatal rat brains as BMECs by immunofluorescence staining of Factor VIII (Unger et al., 2002). Using the *in vitro* primary BMECs cell model and an *in vivo* rat model of ICH, we aimed to explore the potential mechanisms of miR-126-3p in alleviating the complications of ICH such as BBB damage, neuronal damage, and brain edema.

## Materials and Methods

### Patients

Thirty-eight patients with acute spontaneous ICH, ≥18 years of age, and admitted within 48 h after onset were included in this study. Blood samples were drawn upon admission and the serum samples were frozen for later examination. Serum samples from 22 age-matched healthy subjects were used as control. Since the data on expression levels of miR-126-3p in human serum were retrospectively analyzed, written informed consents were waived, and this study (approval No. 2019PS169K) was approved by the Human Ethical Committee of Shengjing Hospital, China Medical University.

### Animal Models of ICH

Adult male Wistar rats (*n* = 48; weighing 250–280 g) were obtained from Charles River Laboratories (Beijing, China). All experiments were carried out according to the National Institutes of Health Guide for the Care and Use of Laboratory Animals. All experiments involving rats were approved (approval no. 2017PS146K) by the Institutional Animal Care and Use Committee of Shengjing Hospital, China Medical University. After anesthetization by injection of 10% chloral hydrate, the rats were placed in a prone position and fixed in a stereotaxic frame. A burr hole (1 mm) was made using a dental drill (0.2 mm anterior to the bregma and 3 mm right lateral to midline, and 6 mm depth below the skull surface). Type VII collagenase was injected at 0.4 μl/min (0.5 U in 2 μl normal saline) into the hole to induce ICH (*n* = 36 rats), as previously described (Rosenberg et al., 1990). The skull of the sham-operated animal was drilled at the same point and an equal volume of saline without collagenase was injected (*n* = 12 rats).

Immediately after modeling, the 36 rats with ICH were randomly divided into three groups: the untreated ICH group, the miR-NC group, and the miR-126-3p group (*n* = 12/group). A 1-mm burr hole was drilled at another position (0.8 mm posterior to the bregma and 1.5 mm right lateral to midline, and 4.5 mm depth below the skull surface). The rats were injected with 10 μM miR-126-3p mimic [5′-UCGUACCGUGAGUAAUAAUGCG-3′ (forward)], and [5′-CAUUAUUACUCACGGUACGAUU-3′ (reverse)] or negative control [miR-NC, 5′-UUCUCCGAACGUGUCACGUTT-3′ (forward)] and [5′-ACGUGACACGUUCGGAGAATT-3′ (reverse)] (GenePharm, Shanghai, China) (5 μl, 0.5 μl/min) (Xi et al., 2017), respectively. The Sham group and the ICH group were treated with the same amount of transfection reagent Entranster^TM^-*in vivo* (Engreen, Beijing, China). After surgery and treatments, the rats recovered for 24 h and were allowed free access to food and water.

### Dual Luciferase Reporter Assay

Vascular cell adhesion molecule 1 wild-type or mutant 3′UTR fragment was subcloned into pmirGLO (Promega, Madison, WI, United States). Until reaching 70% confluence, the cells were starved for 12 h and then co-transfected with 75 pmol miR-126-3p mimic or NC mimic and 1.5 μg recombinant plasmid containing wt or mut 3′UTR. Forty-eight hours post-transfection, luciferase activities were determined by Dual-luciferase reporter assay kit (KeyGen, China).

### Blood Sampling and Brain Tissue Collection

The rats were euthanized and the blood was drawn from the inferior vena cava. The serum samples were frozen for later determination of miR-126-3p expression. The brain was divided into ipsilateral and contralateral hemispheres in relation to ICH. The perihematomal zone was defined as a 2-mm margin around the hematoma. The perihematomal and hematomal tissues were excised, snap-frozen in liquid nitrogen, and stored at −80°C.

### Isolation of BMECs, Cell Culture, Identification, and Transfection

The brains of neonatal rats were isolated and stored in cold PBS (phosphate buffered saline). The cerebellum, diencephalon, pia mater, meningeal blood vessels, and white matter were removed. The cortex was collected, washed with PBS, minced, digested with 0.1% type 2 collagenase (Biosharp, Hefei, China) at 37°C, and filtered through a mesh filter. The mixtures were centrifuged, washed with 20% BSA and digested with a mixture of collagenase and neutral protease (Solarbio, Beijing, China). The cell suspension was added into Percoll (Solarbio) and centrifuged at 1,000 × g for 20 min. BMECs were obtained and cultured in DMEM (Gibco; Thermo Fisher, Waltham, MA, United States) containing 10% FBS (HyClone; GE Healthcare, Logan, UT, United States). Immunofluorescence was used for BMECs identification. The cells were digested with 0.25% EDTA trypsin, blown into single cell suspensions, inoculated into 24-well plates and cultured for 24 h. After removing the coverslips, washing the cells three times with PBS, and removing the floating dead cells, the rat BMECs were seeded into 6-well plates and cultured in serum-free DMEM for 1 h upon reaching 70% confluence. The cells were transfected with 100 pmol of anti-miR-126-3p (5′-CGCAUUAUUACUCACGGUACGA-3′) or anti-miR-NC (5′-CAGUACUUUUGUGUAGUACAA-3′) using Lipofectamine^TM^ LTX Reagent and PLUS^TM^ Reagent (Invitrogen, Carlsbad, CA, United States) according to the manufacturer’s instruction (*n* = 6 BMECs/group).

### BMECs Permeability Assay

BMECs were seeded into Transwell chamber (Costar, United States) at a density of 1 × 104 cells/chamber. After cells were cultured for 72 h, the medium was discarded and 0.01% FITC-dextran 20 (TdB, Sweden) was added into the upper chamber. The medium in the lower chamber was collected at different time points (0, 30, 60, and 120 min after TdB was added). The fluorescence intensity was determined by a fluorescence microplate reader (M200 PRO, Tecan, Switzerland). All measurements were performed in triplicates.

### Western Blotting

Brain tissues were lysed and the samples were centrifuged at 1000 × g for 10 min. BCA assay (Beyotime, Shanghai, China) was performed to determine protein concentration. Forty micrograms of protein samples were subjected to SDS-PAGE and transferred to PVDF (polyvinylidene difluoride) membranes (Millipore). The membranes were incubated with primary antibodies against VCAM-1 (1:400 dilution) (Bioss, Beijing, China), overnight at 4°C. Horseradish peroxidase (HRP)-labeled goat anti-rabbit IgG (1:5000 dilution) or HRP-labeled goat anti-mouse IgG (1:5000 dilution) was used as the secondary antibody. Then, the membranes were incubated with ECL (enhanced chemiluminescence) reagent and exposed in a dark room. Optical densities of the blots were analyzed using the Media Cybernetics Gel-Pro Analyzer software (Rockville, MD, United States). All measurements were performed in triplicates.

### Real-Time Quantitative PCR (RT-qPCR)

The total RNA from the serum samples (humans and rats), brain tissue specimens, and BMECs were extracted using Trizol (Invitrogen, Carlsbad, CA, United States). After determination of the RNA concentration using a Nanodrop2000 device (Thermo Fisher Scientific, Waltham, MA, United States), 1 μg of total RNA was reversely transcribed into cDNA using the PrimeScript RT reagent kit with gDNA Eraser Kit (Takara Bio Inc., Otsu, Japan). DNA removal was conducted at 42°C for 2 min with the addition of 5 × DNA Eraser Buffer and gDNA Eraser. RT-qPCR was carried out on an ABI 7500 quantitative PCR instrument (Applied Biosystems, Foster City, CA, United States) with the SYBR^®^ PremixExTaq (TliRNaseHPlus) kits (Takara Bio Inc., Otsu, Japan). The amplification conditions were: 10 min at 95°C; 10 s at 95°C, 20 s at 60°C and 30 s at 72°C (40 cycles). U6 was used as the internal reference for miR-126-3p. Glyceraldehyde-3-phosphate dehydrogenase (GAPDH) was used as the internal reference for the other mRNAs. The 2^–Δ^ ^Δ^ ^Ct^ method was used to calculate the expression of the target gene in the experimental group and the reference group (Livak and Schmittgen, 2001). The primers were: looped reverse transcription primer, 5′-GTTGGCTCTGGTGCAGGGTCCGAGGTATTCGCACCAGA GCCAACCGCATT-3′; miR-126-3p, 5′-GCATCGTCGTACCGT GAGTAAT-3′ (forward) and 5′-GTGCAGGGTCCGAGGTA TTC-3′ (reverse); U6, 5′-CTCGCTTCGGCAGCACA-3′ (forward) and 5′-AACGCTTCACGAATTTGCGT-3′ (reverse); VCAM-1, 5′-GAAATGACCTTCATCCCTAC-3′ (forward) and 5′-GCTGACCAAGACGGTTGTAT-3′ (reverse); GAPDH, 5′-CCCACTCCTCCACCTTTGAC-3′ (forward) and 5′-ATGAGGTCCACCACCCTGTT-3′ (reverse). The primers were synthesized by Gemma Pharmaceutical Technology Co., Ltd. (Shanghai, China). All measurements were performed in triplicates.

### Statistical Analysis

Statistical analyses were performed using SPSS 19.0 (IBM, Armonk, NY, United States) and GraphPad Prism 6.0 (GraphPad Software Inc., San Diego, CA, United States). Tests for normal distribution (Kolmogorov-Smirnov test) and homogeneity of variance (Levene’s test) were conducted. The data with normal distribution were expressed as means ± the standard error of the mean (SEM) and analyzed by the Student *t* test (two groups) or one-way analysis of variance (ANOVA) with the Tukey *post hoc* test (more than two groups). The data with skewed distribution were tested by non-parametric tests. A *P* < 0.05 was considered statistically significant.

## Results

### The Level of miR-126-3p Is Down-Regulated in Serum of Patients With ICH

**FIGURE 1:**
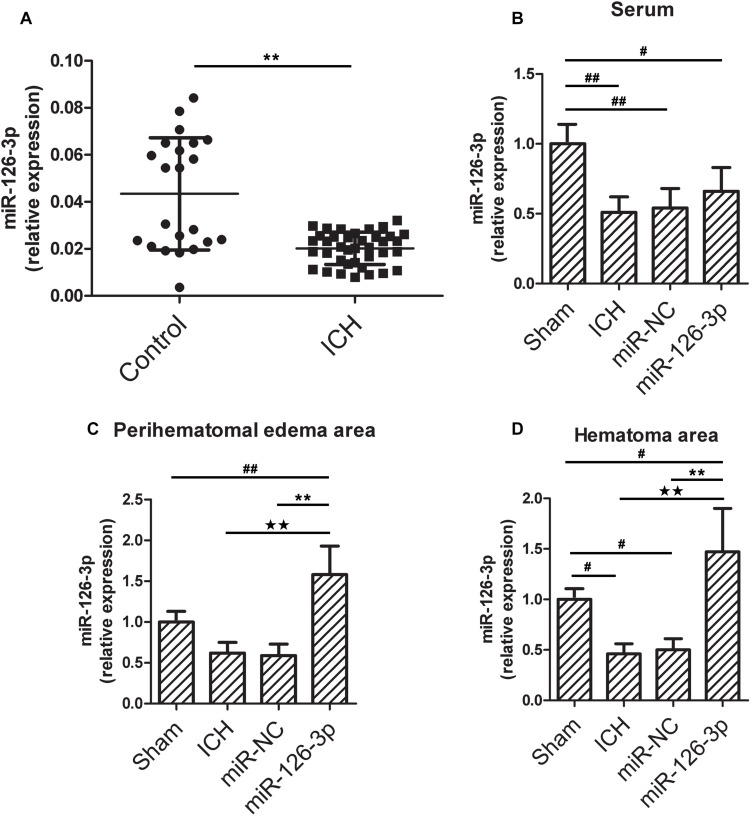
The miR-126-3p levels in serum from patients with ICH and in serum, perihematomal edema, hematoma areas in a rat model of ICH. **(A)** The levels of miR-126-3p in the serum of ICH patients (*n* = 38, within 48h after onset) and healthy subjects (*n* = 22) were determined by RT-qPCR. **(B–D)** Twenty-four hours post-ICH, the levels of miR-126-3p in serum **(B)**, perihematomal edema **(C)**, and hematoma **(D)** areas were measured by RT-qPCR (*n* = 12 per group). All measurements were performed in triplicates; values are indicated with error bars as mean ± SEM. ^#^*P* < 0.05 and ^##^*P* < 0.01 versus Sham group; ^∗∗^*P* < 0.01 versus Control group, miR-NC group, ^★★^*P* < 0.01 versus VCAM-1-wt + control mimics group.

To verify previous miRNA array data (Zhu et al., 2015), we measured the levels of miR-126-3p in serum from ICH patients and healthy subjects by RT-qPCR. Compared with healthy subjects, the level of serum miR-126-3p in ICH patients was 53.4% lower (*P* = 0.0072, ICH group vs. Control group) (Figure 1A). This result confirmed the reduced levels of miR-126-3p in the serum of ICH patients.

### The Transcription Expression Levels miR-126-3p in Serum, Edema Area, and Hemorrhagic Area of the ICH Rats

Next, we explored whether a similar decline of miR-126-3p levels could be detected in the rat model of ICH. As shown in Figures 1B,C, ICH significantly decreased the level of miR-126-3p expression in serum (*P* = 0.0092, ICH group vs. Sham group) and hemorrhagic area (*P* = 0.013, ICH group vs. Sham group) in rats. Whereas, the treatment with miR-126-3p mimic significantly increased miR-126-3p levels in rat brain edema area (*P* = 0.0004, miR-126-3p group vs. miR-NC group) and hemorrhagic area (*P* = 0.0026, miR-126-3p group vs. miR-NC group) (Figures 1C,D). However, no significant increase of serum miR-126-3p was observed in rats administrated with miR-126-3p mimic, compared with the rats treated with the miR-NC (Figure 1B).

### VCAM-1 Is a Direct Target of miR-126-3p

**FIGURE 2:**
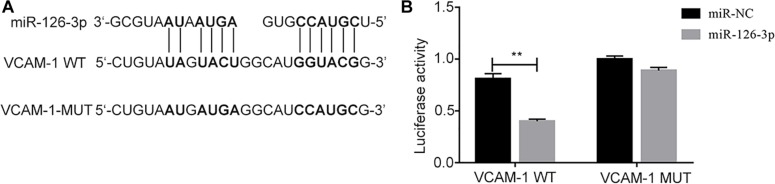
Targeted binding between miR-126-3p and VCAM-1. **(A)** The prediction of the combination between miR-126-3p and VCAM-1 were by TargetScan. **(B)** The targeted combination was validated by dual luciferase reporter assay (*n* = 3 per group). The measurement was performed in triplicates; values are indicated with error bars as mean ± SEM. ^∗∗^*P* < 0.01 versus ICH group.

The binding site of miR-126-3p in the 3′UTR of VCAM-1 was predicated by TargetScan (Figure 2A), and verified by dual luciferase reporter assay. The results showed that compared with VCAM-1-wt + control mimics group, the relative luciferase activity of cells in VCAM-1-wt + miR126-3p mimics group decreased significantly (*P* = 0.0002) (Figure 2B), there was no significant difference in relative fluorescence activity between VCAM-1-mut + control mimics group and VCAM-1-mut + miR126-3p mimics group (*P* > 0.05) (Figure 2B). It showed that there is a targeting relationship between miR126-3p and VCAM-1.

### The ICH Rats Administrated With the miR-126-3p Mimics Have Down-Regulated Expression of VCAM-1 in Brain Perihematomal Tissues

**FIGURE 3:**
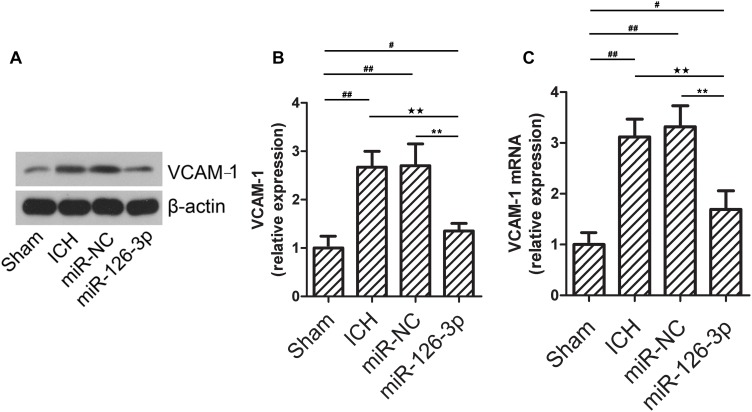
The expression of VCAM-1 in the perihematomal area is down-regulated in the rat with ICH and administrated with miR-126-3p mimics. **(A,B)** The relative protein expression levels of VCAM-1 in the perihematomal tissues of ICH rats were measured by western blotting. β-actin served as an internal control (*n* = 12 per group). **(C)** The relative mRNA expression levels of VCAM-1 in the perihematomal tissues of ICH rats were measured by RT-qPCR (*n* = 12 per group). All measurements were performed in triplicates; values are indicated with error bars as mean ± SEM. ^#^*P* < 0.05, ^ ##^*P* < 0.01 versus Sham group; ^∗∗^*P* < 0.01 versus miR-NC group, ^★★^*P* < 0.01 versus ICH group as denoted.

Vascular cell adhesion molecule 1, an endothelial-specific marker, can be regulated by abnormally expressed miR-126-3p in brain endothelial cells in ICH-injured brain tissues (perihematomal area) (Yousef et al., 2019). To explore the changes of VCAM-1 in ICH and the interaction between miR-126-3p and VCAM-1. We determined whether the expression levels of VCAM-1 were changed in the perihematomal tissues of rat brains by western blotting. As shown in Figures 3A,B, ICH induced a significant elevation of VCAM-1 protein expression in the perihematomal area (*P* = 0.0011, ICH group vs. Sham group). Remarkably, the presence of miR-126-3p mimic significantly reversed the low expression of VCAM-1 caused by ICH (*P* = 0.0037, miR-126-3p group vs. miR-NC group) (Figures 3A,B). Consistently, RT-PCR analyses also indicated that the mRNA expression levels of VCAM-1 had a similar pattern of change as its protein expression levels among the four groups (*P* = 0.0056, ICH group vs. Sham group; *P* = 0.0039, miR-126-3p group vs. miR-NC group) (Figure 3C).

### Validation of Rat BMECs by Immunofluorescence Staining of Factor VIII

**FIGURE 4:**
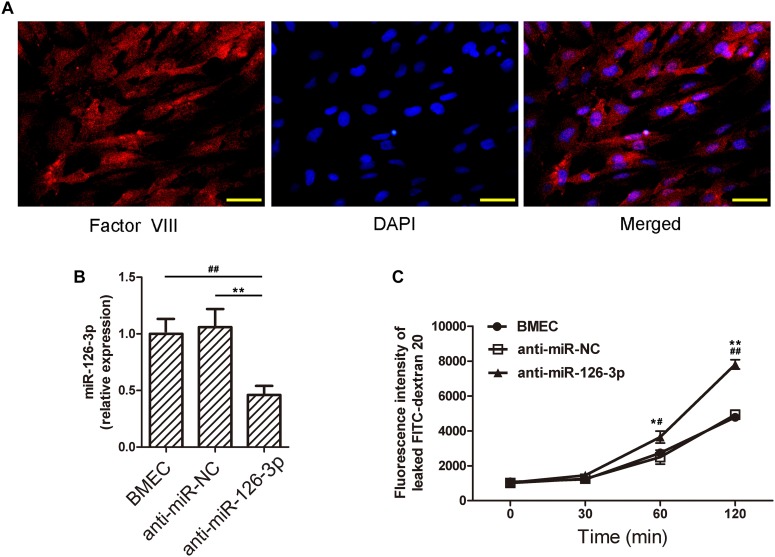
Inhibition of miR-126-3p by anti-miR-126 reduces the levels of miR-126-3p and causes membrane permeability in BMECs. **(A)** Identification of the isolated cells as BMECs. Images show the fluorescence staining of Factor VIII (green), and cell nuclei were stained with DAPI (blue). Scale bar = 50 μm. **(B)** Rats BMECs were isolated and transfected with anti-miR-NC or anti-miR-126. The levels of miR-126-3p were examined by RT-qPCR 24 h after transfection (*n* = 6 per group). **(C)** The permeability of FITC-dextran 20 through a confluent BMECs layer was measured over 120 min (*n* = 6 per group). All measurements were performed in triplicates; values are indicated with error bars as mean ± SEM. ^∗^*P* < 0.05 and ^∗∗^*P* < 0.01, both versus the anti-miR-NC group; ^#^*P* < 0.05 and ^##^*P* < 0.01, both versus the untreated BMEC group.

Factor VIII is a marker generally used to identify human BMECs (Unger et al., 2002). We validated the isolated cells as BMECs by performing immunofluorescence staining for Factor VIII (Figure 4A). The isolated BMECs were used as an *in vitro* BBB model for the subsequent experiments. The miR-126-3p levels in BMECs treated with the anti-miR-126-3p were significantly decreased compared to the cells treated with the anti-miR-NC (*P* = 0.0034, anti-miR-126-3p group vs. anti-miR-NC group) (Figure 4B). This indicates that the transfection procedure of anti-miR-126-3p and anti-miR-NC were successful and that the transfected BMECs could be further used to study the related molecular mechanisms. Notably, our FITC-dextran 20 permeability experiments demonstrated that the inhibition of miR-126-3p by treatment with anti-miR-NC resulted in an obvious BMEC barrier leakage (*P* = 0.021_(__60__min)_ and *P* = 0.0023_(__120__min)_, anti-miR-126-3p group vs. BMEC group) (Figure 4C).

### Inhibition of miR-126-3p Impairs BMECs Barrier Permeability and Up-Regulates VCAM-1 Expression Levels *in vitro*

**FIGURE 5:**
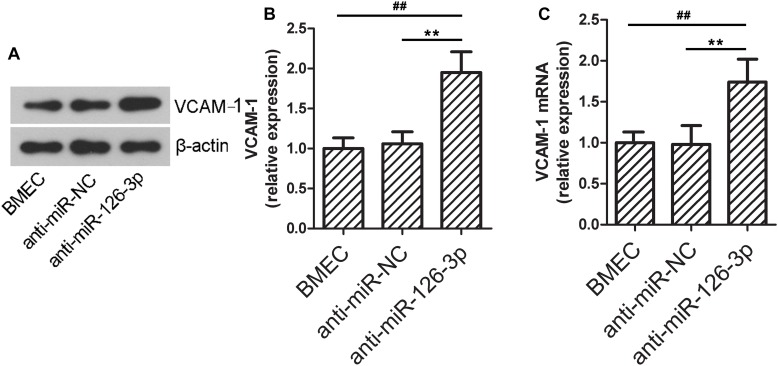
*In vitro* down-regulation of miR-126-3p increases the expression of VCAM-1 in BMECs. **(A,B)** The relative protein expression levels of VCAM-1 in BMECs were determined by western blotting 48 h after transfection of indicated anti-miRNAs. β-actin served as an internal control (*n* = 6 per group). **(C)** The relative mRNA expression levels of VCAM-1 in BMECs were measured by RT-qPCR 24 h after transfection of indicated anti-miRNAs (*n* = 6 per group). All measurements were performed in triplicates; values are indicated with error bars as mean ± SEM. ^∗∗^*P* < 0.01 versus anti-miR-NC group; ^##^*P* < 0.01 versus the untreated BMEC group.

To further validate the role of the miR-126-3p/VCAM-1 axis in BBB function, we tested the effects of *in vitro* miR-126-3p inhibition on VCAM-1 expression in BMECs. As shown in Figures 5A,B, the protein expression of VCAM-1 in the anti-miR-126-3p group was significantly up-regulated compared to the anti-miR-NC group (*P* = 0.0045, anti-miR-126-3p group vs. anti-miR-NC group), as a result of miR-126-3p inhibition. Similarly, the relative mRNA expression levels of VCAM-1 among the three groups were consistent with the patterns of protein expression levels (*P* = 0.0051, anti-miR-126-3p group vs. anti-miR-NC group) (Figure 5C).

## Discussion

Intracerebral hemorrhage is a common type of stroke and is among the leading causes of death in the world (Qureshi et al., 2009). The expression of circulating miRNAs vary with different stroke subtypes, and ICH causes the obvious changes in expression levels of miRNA in brain tissue and blood in a unique pattern (Martinez and Peplow, 2017). Several studies have reported changes in the expression of miRNAs in the brain of stroke patients compared with healthy people. These studies have shown that miRNAs are pathogenic and pathological intermediate products in ICH (Tan et al., 2011). Therefore, circulating miRNAs could be used as stroke biomarkers. In the present study, we first validated the down-regulation of miR-126-3p in the serum of patients with ICH. Then, we focused on the exploration of the related mechanisms involved in the protective effects of miR-126-3p in ICH. Based on previous studies (Harris et al., 2008; Bai et al., 2017), we predicted that miR-126-3p might target VCAM-1. We validated the increased level of VCAM-1 expression and decreased level of miR-126-3p transcript in brain hemorrhagic area of rats with ICH. In an *in vitro* primary rats BMECs model that simulates BBB, through manipulation of miR-126-3p expression by treatments with miR-126-3p mimics and anti-miR126 in BMECs, we confirmed that miR-126-3p could alleviate BBB disruption, which was associated with targeted regulation of VCAM-1.

Many recent mechanistic studies have revealed the role of different miRNAs in the pathogenesis of ICH. For example, the down-regulation of miR-181b has been shown to increase HSP5A, which is involved in reticulum endoplasmic stress, resulting in secretion of inflammatory cytokines, brain edema, and neurological injury (Wang et al., 2018). In addition to their diagnostic uses, miRNA could also be used in the treatment of ICH (Jeyaseelan et al., 2007). The over-expression of miR-144-3p aggravates neurological injury after ICH through neuronal apoptosis by the PI3K/AKT pathway (Fan et al., 2018). Moreover, miR-23a-3p has been reported to promote edema formation after ICH via ZO-1 (Hu et al., 2018). On the other hand, miR-21, miR-27a-3p, and miR-590-5p have been unraveled to exert neuroprotective effects after ICH (Guo et al., 2018; Xi et al., 2018; Zhang et al., 2018). Hence, miRNAs play a wide variety of roles after ICH, and through a number of mechanisms. A previous study by our group strongly suggests that miR-126-3p has a potential therapeutic effect on brain injury after ICH (Xi et al., 2017). Two types of miRNA related therapeutics are currently in exploration: miRNA mimics (miR-mimic) and miRNA inhibitors (antagomir) (Xu et al., 2018). miRNA mimics can restore the loss of beneficial miRNAs, while antagomirs help deplete detrimental miRNAs. Although the *in vitro* use of a miR-mimic or antagomir is a well-established method to identify miRNA functions, so far only a few of them have been successfully applied in clinical.

The BBB is composed of pericytes, astrocytes, endothelial cells, basement membrane, and extracellular matrix. The BBB can resist harmful components from the blood to enter into brain tissues and protect the brain from invasion (Bertrand et al., 2016). Inflammation directly destroys the BBB, resulting in brain edema (Schlunk et al., 2016). We successfully isolated BMECs and used immunofluorescence staining to verify the presence of factor VIII (Unger et al., 2002). Then, we cultured these cells and obtained a monolayer of cell membrane structure. Subsequent experiments with FITC-dextran 20 verified that miR-126-3p regulated the permeability of this BBB structure, as inhibiting miR-126-3p with anti-miR-126 significantly increased the permeability of the monolayer membrane. Therefore, it is plausible that the decrease of miR-126-3p may also lead to the destruction of the BBB *in vivo*, resulting in further tissue damage.

VCAM-1 is a target gene of miR-126 (Harris et al., 2008). Studies have shown that miR-126 inhibits vascular inflammation and promotes angiogenesis in rat models of spinal cord injury by reducing the expression of VCAM-1 (Hu et al., 2015). The leukocyte inflammatory chemotaxis process is inseparable from the regulation of adhesion molecules expressed by endothelial cells (Nieto-Lima et al., 2018). First, the rotational movement of leukocytes is mediated by a selection and its glycoprotein ligands (Luo et al., 2007). Secondly, leukocytes and endothelial cells are activated by cytokines and chemokines, promoting the expression of adhesion molecules and integrin ligands, which in turn mediate the tight leukocyte adhesion and binding to endothelial cells (Salmi and Jalkanen, 2005). Eventually the leukocytes break through the endothelial barrier and reach lesion site. Under normal physiological conditions, endothelial cells are inactive and do not express adhesion molecules, but under pathological conditions (such as BBB damage), a large number of exogenous cytokines are generated and the endothelial cells are activated, so that VCAM-1 is expressed (Park et al., 2018). VCAM-1 on the surface of endothelial cells mediates leukocyte adhesion through its interaction with VLA-4, which consists of CD49d (α4) and CD29 (β1). VLA-4 is expressed on most leukocytes and activated neutrophils (Tissino et al., 2018). Although endothelial cells do not express VCAM-1 normally, cytokines or bacterial products can stimulate endothelial cells to express VCAM-1 within 4–12 h. It is generally believed that the expression of VCAM-1 is regulated at the initial transcription stage, and a series of transcription factors regulate the expression of VCAM-1 through the interaction between them and its interaction with the VCAM-1 gene (Davel et al., 2017).

Recently, studies have confirmed that miR-126 in endothelial cells can inhibit the expression of VCAM-1 (Harris et al., 2008), therefore, regulating endothelial cell adhesion and vascular inflammation. In addition, it has been recently demonstrated that the expression of miR-126 is stimulated by blood flow and is regulated by the zinc finger transcription factor klf2a (Nicoli et al., 2010; Mondadori dos Santos et al., 2015). The present study serves as a proof-of-concept investigation showing that the miR-126-3p mimic reduces the expression of VCAM-1 following ICH *in vivo*. This indicates that miR-126-3p can reduce the degree of cerebral hemorrhage by targeted inhibition of VCAM-1. The *in vitro* blocking of miR-126-3p results in the up-regulated expression of VCAM-1 protein and mRNA, suggesting that miR-126-3p directly inhibits the expression of VCAM-1. The reduction of miR-126-3p following ICH is probably directly related to the up-regulated expression of VCAM-1 and exacerbated infiltration of inflammatory cells. Nevertheless, VCAM-1 is probably not the only molecule that is responsible for the functional loss of BBB. Additional studies are required to further elucidate the exact mechanisms and other actors involved.

The dysregulation of miRNAs in ICH suggest that miRNAs may be used as biomarkers for the diagnosis and prognosis of ICH (Martinez and Peplow, 2017). The regulatory network of miRNAs and their target genes is very complicated. Therefore, understanding the mechanisms of the occurrence and development of ICH depends on a more comprehensive understanding of the role of different miRNAs in the disease. A more in-depth understanding of miRNAs can also reveal potential pharmacological targets for new drugs to promote the treatment of ICH. The current investigation has several weaknesses and limitations. First, future studies will be needed to examine the effects of the miR-126-3p mimic in animals without ICH, which could reflect the potential toxcicity of the miR-126-3p mimic. Second, the effect of miR-126-3p over expression on BMECs permeability or VCAM-1 expression should be verified in the future. Third, due to ethical issues, we only measured the levels of serum miR126-3p in patients with ICH. Even though this experiment was somehow representative, it would be more convincing if brain tissue specimens from ICH patient were collected during surgery.

## Conclusion

We confirmed that serum miR-126-3p level is lower in patients with ICH than that in healthy control subjects. The *in vivo* administration of miR-126-3p minics in the rat ICH model and *in vitro* treatment of BMECs with anti-miR126 alleviated BBB disruption, which was demonstrated to be associated with VCAM-1 regulation. Our work suggests that the miR-126-3p/VCAM-1 axis is a potential therapeutic target for patients with ICH.

## Data Availability

The datasets generated for this study can be found in GenBank (BankIt2245039 rno-miRNA-126-3p MN238920).

## Ethics Statement

This study was approved by the Institutional Review Board and the Institutional Animal Care and Use Committee of Shengjing Hospital, China Medical University (approval no. 2017PS146K). The procedures on the animals complied with the Guide for the Care and Use of Laboratory Animals by the National Institutes of Health (NIH Publication No. 80-23). The animal experiments adhered to the principle of using the least number of animals strictly to complete the experiments with minimized pain of experimental animals. Since the data on expression levels of miR-126-3p in human serum were retrospectively analyzed, written informed consents were waived, and this study was approved by the Human Ethical Committee of Shengjing Hospital, China Medical University (approval no. 2019PS169K).

## Author Contributions

XF wrote the manuscript and researched the data. TN contributed to discussion. XL designed the study, reviewed the data, and revised the manuscript. All authors read and approved the final manuscript.

## Conflict of Interest Statement

The authors declare that the research was conducted in the absence of any commercial or financial relationships that could be construed as a potential conflict of interest.

## Abbreviations

BBB: blood-brain barrier
BMECs: brain microvascular endothelial cells
EGFL7: epidermal growth factor-like-domain 7
ICH: intracerebral hemorrhage
NVU: neurovascular unit
VCAM-1: vascular cell adhesion molecule 1.
